# Industrial pipelines data generator

**DOI:** 10.1016/j.dib.2020.106275

**Published:** 2020-09-03

**Authors:** Mubarak AL-Alawi, Ahmed Bouferguene, Yasser Mohamed

**Affiliations:** a Department of Civil and Architectural Engineering, Sultan Qaboos University, Seeb, Oman; b Department of Civil and Environmental Engineering, University of Alberta, 116 St & 85 Ave, Edmonton, Canada, U.S.A

**Keywords:** Industrial Pipelines, Random, Optimization, Construction, Markov chain, Validation

## Abstract

The construction of industrial projects involves fabricating and installing massive quantities of pipelines. The design data of pipelines is complex, usually unique for each project and not easily available to researchers and the public for confidentiality reasons. As a result, an industrial pipelines data generator able to realistically simulate pipelines structures will lessen the dependence on real-life data. This article describes an industrial pipelines data generator that was developed using topological and physical properties of pipelines from real industrial projects. This generator can simulate the properties (the type of component, length, diameter, running direction, and the connectivity relationships between components) of real industrial pipelines. Its application in construction engineering and management research, more specifically in the experimental analysis of an optimization algorithm, was described by the authors in previous work [1]. The data generator can be used to develop benchmark problem instances for optimization problems and for simulation studies of construction operations in industrial projects.

## Specifications Table

**Table:** 

Subject	Construction Engineering and Operational Research
Specific subject area	Dataset generation for construction engineering optimization problems
Type of data	Text Program code
How data were acquired	Industrial pipelines data generator is developed using Markov Chain model. Real industrial pipelines data are used in developing the industrial pipelines data generator. The generator produces industrial pipelines data similar to real pipelines.
Data format	Raw data
Parameters for data collection	Real industrial pipelines data was used to build the industrial pipelines data generator
Description of data collection	An industrial pipelines project database was analysed. All pipelines formation components properties were studied and the relationships among them were identified. The generated data was validated by comparing it against real industrial pipelines data.
Data source location	Institution: University of Alberta City/Region: Edmonton/Alberta Country: Canada
Data accessibility	Repository name: Mendeley Data DOI: 10.17632/68c4c77f4s.3 Direct URL to data: http://dx.doi.org/10.17632/68c4c77f4s.3
Related research article	

## Value of the Data

• The proposed industrial pipelines data generator allows simulating realistic industrial pipelines data structures. As a result, this generator can alleviate the need for real-life project data which generally are not publically available.
• Thanks to the ease of generating simulated (yet realistic) industrial pipeline datasets, researchers can rapidly start exploring and testing their ideas in order to gain better understanding about the amount and the type of real data that need to be collected for validation.
• With this generator, researchers will be able to generate replicates of a simulated (yet realistic) reference project, which in practice is impossible to do. This allows statistical analyses to be conducted with respect to fabrication, transportation, on-site installation processes, etc.
• Since this generator allows researchers to have access to similar pipeline datasets, it becomes possible to conduct statistical comparisons of the efficiency of optimization algorithms including those used to design 3D pipeline structures that are constrained to fit in a pre-defined volume.

## 1 Data Description

**Fig. 1:**
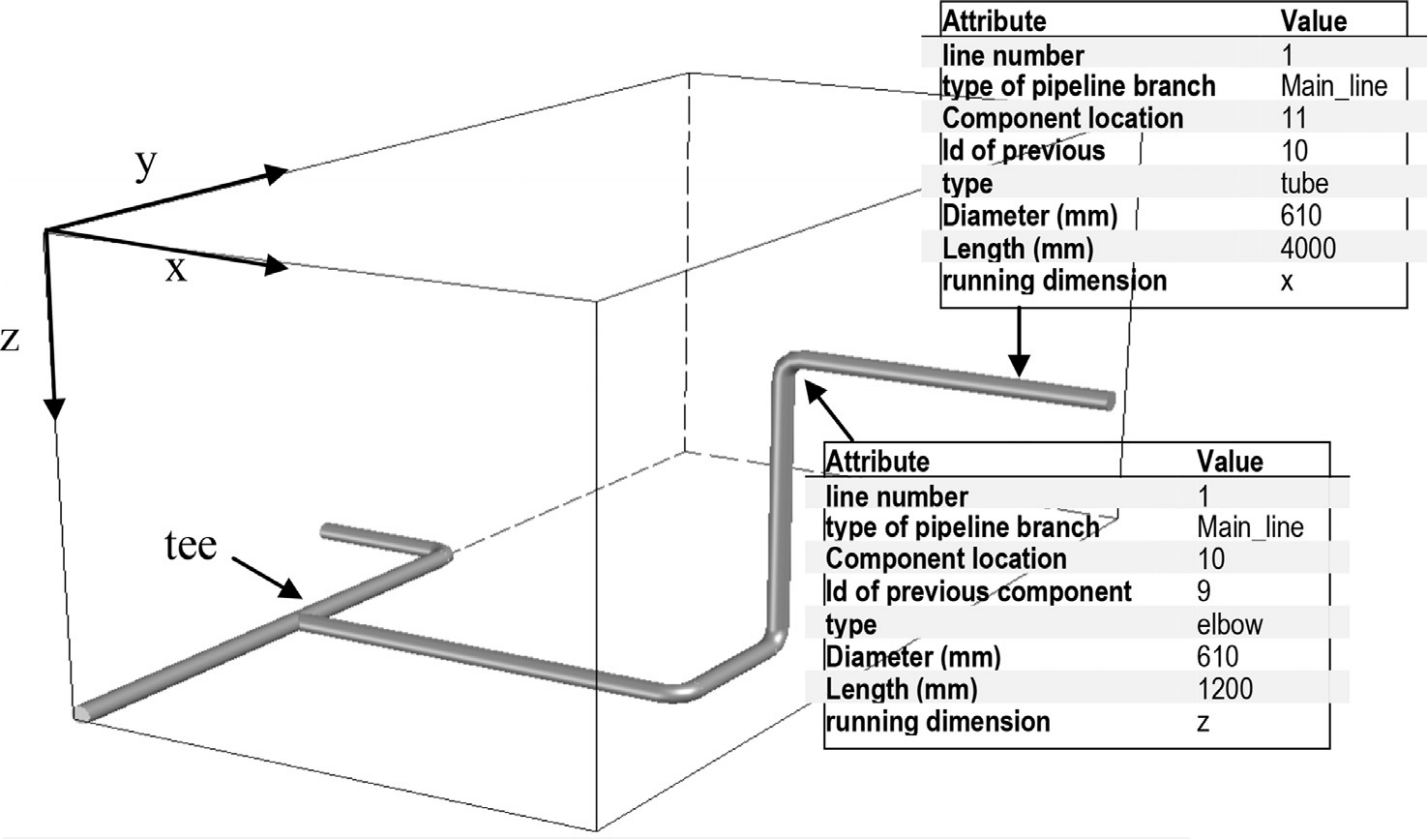
Section of an industrial pipeline

The industrial pipelines data generator generates a set of pipelines data, which consists of a list of connected components. Each component has eight attributes as demonstrated in Fig. 1. These are: (1) line number, (2) type of pipeline branch, (3) component location (seq_in_branch), (4) id of the previously connected component, (5) component type, (6) component diameter, (7) component length, and (8) running direction (x, y, z) of the component. These properties define the physical properties of the pipeline.

**Fig. 2:**
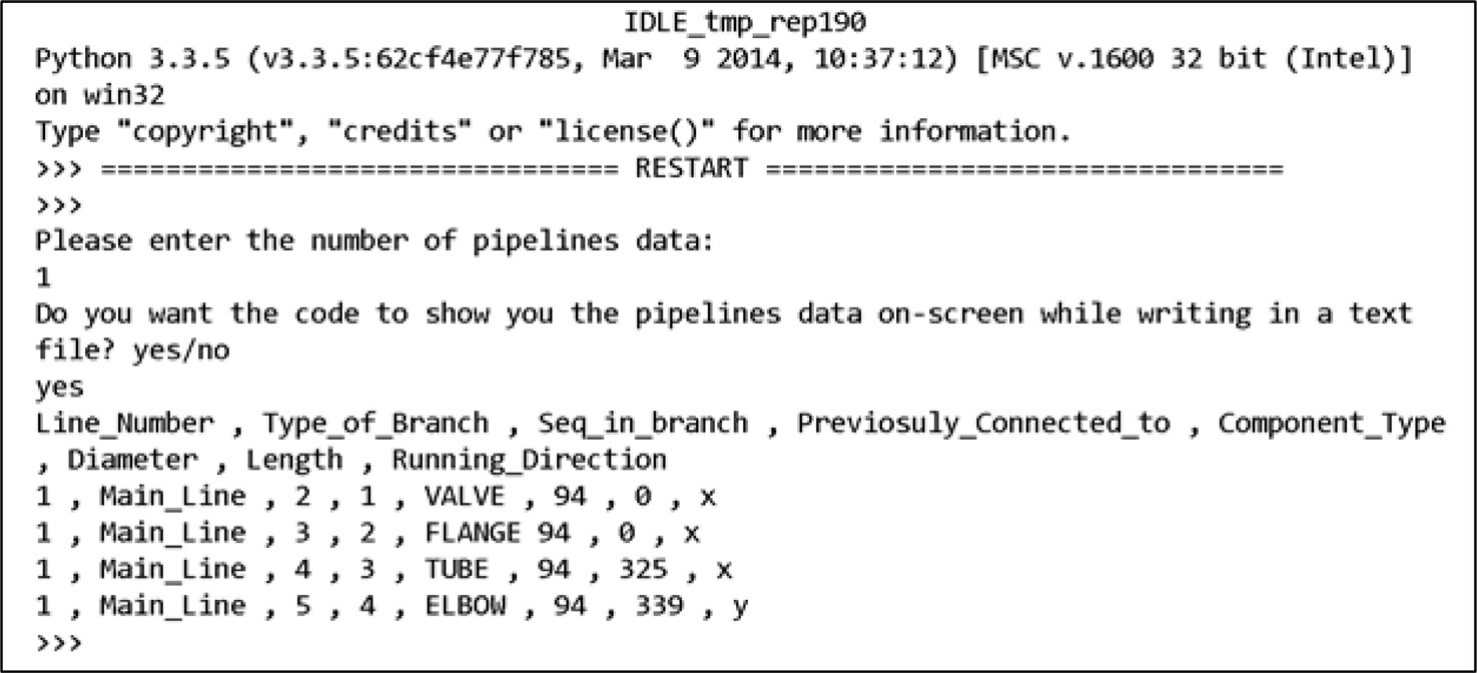
Industrial pipelines data generator user screen

Fig. 2 shows the generator user screen. The program code was written using Python programming language. The program runs in a simple Python Integrated Development Environment (IDLE). The application prompts the user to enter the number of pipeline datasets required and outputs the generated data to a text file located in the same directory as the program code. A set of simulated industrial pipeline structure data are described in Al-Alawi, Bouferguene, and Mohamed [2].

## 2 Experimental Design, Materials and Methods

The data from a real industrial project was used to build the properties of the industrial pipelines data. The total number of pipelines used to build the proposed generator is 1052 with a total of 33324 components. The generated data is organized in a tree structure whose nodes describe the pipeline components. Each node in this tree is a data structure consisting of an array of values like the type of component, length, material etc. The pipelines components are *tube, elbow, flange, tee, valve, fblind, ftube, reducer, closure, cap, instrument, coupling, and pcomponent*.

A recursive function is used to breakdown the pipelines data into two-sub pipelines, the main branch and the secondary branches extending from the main branch. The properties of each branch are investigated and the correlation between different pipelines components are identified (refer to [2]). In order to use the Markov chain model to generate the pipelines data structure, two correlation matrices are used. The Markov chain model is used to realistically generate the sequence of pipelines components and to preserve the realistic topological structure of the pipelines data. Markov chain generates a sequence of future events or variables based on the current state of the system [3]. Practically, the application of the Markov chain model uses two 13 × 13-transition matrices associated with the main and secondary branches, respectively. In addition, since the Markov chain model process must preserve the feature that regulates the re-occurrence of pipelines components, the probability functions describing the occurrence of seven pipeline components (pcomponent, instrument, valve, flange, elbow, tee, and reducer) were identified and joined with the transition matrices to generate realistic sequences of pipeline components. The seven components enumerated above are the only ones needed at this stage since the other ones can only be located at the beginning or at the end of the list of components describing a pipeline.

Once the user enters the number of pipelines *N* to be generated, the generator proceeds to generating these pipelines sequentially according to the following protocol. For each pipeline *P_n_*, the generator starts by outputting the sequence of its components, which constitutes the first layer of operation. This process is broken down into two stages; during the first stage, the components of the main branch are generated and if a tee is encountered in the list of components, the second stage is initiated and proceeds to generating the components for *P_n_* secondary branch. It is important to note that a main branch can have many secondary branches.

The second layer of operation assigns pipeline *P_n_* diameter. For this purpose, the pipeline diameter is randomly selected from a probability distribution function that describes the tube component diameter found in original data. The pipeline diameter will remain constant unless a reducer in encountered in the list of pipeline *P_n_* components. In this case, the generator reduces the diameter to a new value randomly selected from the probability distribution function that describes the reducer diameter found in the original data.

The third layer in the generation process assigns lengths to pipeline *P_n_* components using an approach similar to that adopted for selecting the diameter. The fourth layer in the generation process assigns the running direction (x, y, z) to the pipelines components. This layer replicates the space the pipeline may occupy in reality. Finally, each pipeline components will be presented in the following form:

[Line_number, Type_of_branch, Seq_in_branch, Previously_connected_to, Component_type, Diameter, Length, Running_direction].

The industrial pipeline data generator went through extensive data validation processes (refer to [4]). Evaluation of number of components and correlation analysis, clustering-based analysis, validation of distance between feature vectors were adopted to compare randomly generated pipelines data to real one. The validation process showed that the generated industrial pipelines data resemble real pipelines data.

## Declaration of Competing Interest

The authors declare that they have no known competing financial interests or personal relationships which have, or could be perceived to have, influenced the work reported in this article.

## Appendix Supplementary materials

Image, application 1
